# Roundtable Discussion: Making Sense of Current Liposuction Technologies

**DOI:** 10.1093/asjof/ojab045

**Published:** 2021-11-09

**Authors:** Jonathan Cook, Jason N Pozner, David M Turer, Barry E DiBernardo, Gaurav Bharti, Bill Kortesis, Diane I Duncan

## Abstract

Suction-assisted lipectomy (or “liposuction”) is a fundamental technique for all plastic surgeons, and like many procedures in aesthetic surgery, its applications are continuing to evolve. With the rapid introduction of new technologies, many plastic surgeons are left with questions about how these new devices work, what results to expect, and how to best apply these technologies in their practice. We recognized that there is a need for unbiased recommendations to guide surgeons on how to approach current liposuction devices (as well as their adjuncts) and how to use them effectively for their patients. Using available literature and personal experience, we answer the most common questions that we hear from our plastic surgery colleagues.

Liposuction is one of the most commonly performed surgical procedures in plastic surgery, performed by 89.1% of all ABPS board-certified plastic surgeons in the United States. In 2020, it was the most common aesthetic surgical procedure in the United States, with 296,601 procedures performed. This trend has held true over the past 10 years, with liposuction consistently earning the first or second most common procedure, along with breast augmentation. Liposuction was also the most popular surgical procedure for men in 2020, with 11,588 procedures performed.^1^

Plastic surgeons must be comfortable with the current technologies available to supplement and enhance liposuction, and we present here a round table-style discussion to answer the most common questions we hear about liposuction and associated technologies.

The discussants were Jason N. Pozner, Jonathan Cook, Barry E. DiBernardo, David M. Turer, Bill Kortesis, Gaurav Bharti, and Diane I. Duncan.

## ROUNDTABLE DISCUSSION

**1. What devices do you have in your office**?

**Table:** 

	Dr Pozner and Dr Cook	Dr DiBernardo and Dr Turer	Dr Kortesis	Dr Duncan
Power-Assisted Liposuction (PAL) (MicroAire, Charlottesville, VA)	Yes	Yes	Yes	No
Ultrasound-Assisted Liposuction (UAL) (VASER, Solta Medical, Bothell, WA)	Yes	Yes	Yes	No
Laser-Assisted Liposuction (LAL) (ProLipo, Sciton, Palo Alto, CA/SmartLipo, Cynosure, Westford, MA)	Both	SmartLipo	SmartLipo	Yes, but don’t use
Bipolar Radiofrequency-Assisted Liposuction (bipolar RFAL) (BodyTite, FaceTite, AccuTite; Inmode, Lake Forest, CA)	Yes	Yes	Yes	Yes
Helium Plasma RFAL (Renuvion, Apyx Medical, Clearwater, FL)	Yes	Yes	Yes	Yes
Others:	Arvati (Thermi, Irving, TX)	Arvati, Body Jet (HumanMed, Schwerin, Germany)	Hydrasolve (Andrew Technologies, Los Angeles, CA), but no longer use. Thermi, but no longer use	Thermi, but no longer use

**2. Do you perform liposuction under local anesthesia or general anesthesia (or both)**? **What are the advantages and disadvantages**?

Dr Pozner and Dr Cook:

We use local for smaller areas, and mostly will do only one area (such as the abdomen) at a time. I don’t like high lidocaine levels. Obviously, under general anesthesia, you can do more, and the patient is very comfortable. But with “MKO Melt” sublingual tablets (combination midazolam/ketamine/ondansetron by ImprimisRx [San Diego, CA]), along with patient-administered nitrous oxide (using the ProNox system [CAREstream Medical, Surrey British Columbia, Canada]), administering tumescent fluid (which can be quite uncomfortable under local anesthesia) becomes much better tolerated. Also, the patient can move to better position themselves to help you. For arms, we really prefer local—these cases can be frustrating when working around an anesthesiologist, pulse oximeter, intravenous line, and blood pressure cuff in the way.

Dr DiBernardo and Dr Turer:

We offer liposuction under local anesthesia with ProNox, under IV sedation, or under general anesthesia. We have 3 locations: our office for smaller cases (with local and/or ProNox); our surgery center for medium cases (liposuction of 2-3 areas); and the hospital for large, high volume, or combined cases with either higher risk, medical problems, or patients needing overnight stay for large fluid shifts. We find that the multiple location approach allows us to tailor the staff, anesthesia, facility, and equipment to our patients’ needs and operative requirements.

Dr Kortesis and Dr Bharti:

We prefer general anesthesia because of our multistep liposuction process. Most patients prefer 360° contouring of the body part that we are performing. Therefore, general anesthesia is required because of the area. We will also perform liposuction under local anesthesia as well in certain smaller cases. All procedures are performed in our AAAASF accredited surgery center.

Dr Duncan:

I do most of my “big procedures,” including liposuction, under general anesthesia. I have a class C AAAASF certification. However, many patients frequently request local anesthesia and/or IV sedation, as the perceived risk with general is high and they don’t like the recovery aspect. I am using ProNox and MKO Melt to do small to medium cases, such as a single area of RFAL. Sometimes we may lose a case, if the patient wants local but the case is too big. Ideally, we offer all options: general, IV propofol, IV sedation, local anesthesia with ProNox, or straight local anesthesia alone.

3. **What is your operative approach, with respect to incision placement?**

Dr Pozner and Dr Cook:

For the abdomen and flanks, we place incisions within the “bikini line,” and will mark incisions while the patient is wearing a photographic garment, so that the resulting scars are hidden. For the flanks, we place incisions posteriorly within the “bikini line” so they are not visible to the patient from the front. For arms, we place incisions at the elbow and the axilla, within a natural skin fold. For inner thighs, we place incisions within the groin crease, and for outer thighs, I place incisions at the lateral inferior gluteal crease. For knees, we place incisions at the inner medial knee (this incision is less visible). For upper back (“bra fat”), we place an incision within a natural axillary skin crease. And finally, for the neck, we use a submental incision, within the submental crease.

Dr DiBernardo and Dr Turer:

We use all of the above, and always try to place incisions in natural skin creases, or in areas hidden by clothes whenever possible.

Dr Kortesis and Dr Bharti:

We place abdominal incisions as laterally as possible in the back and intentionally make these slightly asymmetrical. We do not use a midline incision when prone—we tried and used this in the past with poor scaring results. For the abdomen, we place one incision under each breast, one at the umbilicus, and two under the bikini area. For the back, we place one at the midline gluteal cleft, two under the bikini line as lateral as possible, and the same in the bra area. For the arms, we place one within the axilla, and one within the elbow flexion crease. For legs, we prefer the groin crease and posterior buttock crease; we avoid a knee incision if possible (I find these are often too visible).

Dr Duncan:

I have the patient put on a bikini during marking, to ensure that access ports are within the garment edges. I will mirror incisions if they will be covered by clothing but stagger incisions (making them intentionally asymmetrical) if they will be exposed. I do utilize a midline access port posteriorly, if appropriate. I try to optimize access to all areas needing treatment so fewer access ports are needed. In arms, though, I treat circumferentially, so about 5 access ports are needed. Today’s devices are small in diameter, so an 18-gauge needle poke, expanded with narrow lipo scissors, leaves only a tiny mark. I give the patients Strataderm (Stratpharma, Basel, Switzerland) and Silagen (NewMedical Technology, Northbrook, IL) patches postop. Most of the redness is gone in 7 weeks.

4. **What is your operative approach, with respect to patient positioning**?

Dr Pozner and Dr Cook:

We try not to use prone positioning unless we are performing gluteal lipoaugmentation. We do better with positioning the patient supine and in the lateral decubitus positions, especially when performing liposuction on the flanks. We are limited a bit in terms of cannula length by the capacity of our M11 Steam Sterilizer (Midmark, Dayton, OH), so we use more smaller incisions.

Dr DiBernardo and Dr Turer:

We use prone positioning for the upper and lower back, buttocks, and posterior thighs. We do not routinely place a “bump” under the pelvis, but we will use “frog-leg” position for the thighs.

Dr Kortesis and Dr Bharti:

For the trunk, we position the patient supine and prone with chest rolls. For the arms, they must be mobile for me to be able to perform four position liposuction. For the legs, we also position the patient supine and prone for 360° contouring.

Dr Duncan:

I always prone the patient to reach the posterior flanks and back—it saves time. I utilize a bump for the pelvis, or stirrups. I always use “frog leg” position for the thighs, or stirrups again, if appropriate; these provide better access to all regions. Finally, I will place a wedge under the waistline for accessing the thighs and flanks when prone.

5. **What technologies do you prefer for skin tightening**? **What areas are best suited to each device**?

Dr Pozner and Dr Cook:

We prefer BodyTite (FaceTite, AccuTite; Inmode, Lake Forest, CA) and Renuvion (Apyx Medical, Clearwater, FL). We are fans of BodyTite (we recently submitted our experience with using bipolar RFAL on 750 patients to ASJ), and we are still determining how well Renuvion stacks up to BodyTite. For the neck and face—AccuTite (Inmode, Lake Forest, CA) . For men’s chest (ie, treatment of gynecomastia), we use VASER, plus or minus BodyTite or Renuvion.

Dr DiBernardo and Dr Turer:

We use BodyTite, FaceTite, and AccuTite. We also like PrecisionTx (Cynosure, Westford, MA). We have also been using Renuvion on abdominal and large-area liposuction cases. Our preferences for each area: for arms, SmartLipo (Cynosure, Westford, MA); for the neck, FaceTite or AccuTite; for the body, BodyTite or Renuvion; for treating body skin and fat with one device, SmartLipo; for deep “vertical” tightening, Renuvion; and for the breast, ThermiTight (Thermi, Irving, TX) did the most studies, but the FaceTite handpiece works well.

Dr Kortesis and Dr Bharti:

We use Renuvion for large areas such as the trunk and extremities (we like and prefer the fibroseptal reduction, resulting in 3-dimensional tightening). For smaller and finite areas, we prefer BodyTite/FaceTite (ie, around the elbows, knees, necks, and jawlines).

Dr Duncan:

I like Renuvion for large surface areas. It is fast and the results can be seen immediately. The best areas include the flanks and bra roll, primary necks, and the abdomen. BodyTite I use for directional shaping. I do frequently combine the 2 technologies (Renuvion and BodyTite) when treating an especially demanding area.

6. **When do you combine technologies**?

Dr Pozner and Dr Cook:

We use a simple algorithm. We use MicroAire (PAL) (Charlottesville, PA) for young patients with a large amount of abdominal or truncal adiposity; they do not require the skin tightening effect of an energy device. For older patients in this same category (ie, a large amount of abdominal or truncal adiposity) we will combine MicroAire with BodyTite or Renuvion depending upon the area (large areas are faster with Renuvion). For male patients with gynecomastia, we use VASER plus another energy device (either BodyTite or Renuvion). We are starting to trial combination treatments using both Renuvion and BodyTite—so far, we are not sure if there are any advantages to combination treatment, but each device seems to heat a bit differently. The BodyTite in my mind is better for skin tightening (as it applies bipolar RF energy across the dermis), and the Renuvion is better for volumetric tightening (as it targets the fibroseptal network).

Dr DiBernardo and Dr Turer:

We use PAL (MicroAire) or UAL (VASER) combined with bipolar RFAL (BodyTite) for about 90% of our body contouring procedures.

Dr Kortesis and Dr Bharti:

Our preferred methodology includes a very standard approach of: (1) VASER, (2) “Separation” (following Dr Simeon Wall Jr.’s SAFELipo technique),^2,3^ (3) Liposuction (“Aspiration” of SAFE), (4) “Equalization” of fat and feathering (SAFE), and finally (5) Renuvion or BodyTite.

Dr Duncan:

When working in the breast, combining Renuvion and BodyTite gets the most lift. I also combine with suspension threads for creating an internal support framework; I use Double Forte PDO threads (Miracu, Mission Viejo, CA) to fine tune symmetry, especially the level of the NAC. I also use both devices when treating the arms, in moderate to challenging cases. A tip—KT tape (KT Health, American Fork, UT), 2 horizontal pieces placed in the deltoid region postop can improve the appearance of the volar skin, which otherwise tends to become crepey postop. For the abdomen with diastasis, I use Renuvion. This can significantly reduce the diastasis if present. I will make 3-5 passes directly on top of the midline fascia, angling out slightly. The RF energy will immediately cause fascial contraction. While not an abdominoplasty substitute, this therapy in addition to soft tissue tightening is frequently a great tummy tuck alternative. If the skin quality is very lax, I will sometimes add BodyTite to reduce skin crepiness. By using the bipolar head like an iron, the skin tightness can be optimized. I use BodyTite for the same purpose in arms and inner thighs. For the Neck, Renuvion will dramatically improve the suprasternal laxity that some other energy-based devices don’t address as well. The FaceTite is a consistent performer in necks that need mild to moderate tightening. I am quite happy with the AccuTite for microlifting of the jowl and midface. I would not use this device around the eyes or forehead, as I have had one case of facial nerve weakness there posttreatment.

I would not combine all three technologies (PAL, UAL, bipolar RF). PAL plus UAL makes sense. But adding a second heating device after aggressive mechanical suction is not necessary in my view. Risky. I have seen some serious postop complications from aggressive use of three technologies combined, especially in secondary cases. This is an important topic—how much is too much? I recently saw a patient treated this way in a South American clinic. She had a 4 × 7 cm area of full-thickness skin loss on the distal aspect of her inner thigh due to postop ischemia. The patient has some residual woody induration, erythema, and fibrosis in the anterior thigh on the same side. Her facial treatment resulted in a profound marginal mandibular nerve palsy. Her thigh burn had to be excised, leaving her with a permanent 10 cm scar.

7. **What are your approaches to specific anatomic areas (eg, axillary “bra fat,” abdomen and flanks, arms, medial thighs, knees, and lower legs)**?

### Axillary “Bra Fat”

Dr Pozner and Dr Cook:

FaceTite handpiece all the way.

Dr DiBernardo and Dr Turer:

We like SmartLipo here because it is small and can address both the fat and skin tightening together using one device.

Dr Kortesis and Dr Bharti:

No matter which energy device you use, make sure to get all layers of fat and to break down any adhesions to the area. This will allow for adequate skin redraping and smoothing.

Dr Duncan:

RF treats this area incredibly well. The shrinkage of skin can be phenomenal, even in massive weight loss patients. Either Renuvion or BodyTite will work well.

### Abdomen and Flanks

Dr Pozner and Dr Cook:

BodyTite vs. Renuvion—unsure at this point which is better. Renuvion is faster, but BodyTite seems better for skin tightening (as mentioned previously).

Dr DiBernardo and Dr Turer:

BodyTite and VASER are our go-to for most of these.

Dr Kortesis and Dr Bharti:

It is important to either perform adequate separation as part of SAFE liposuction or a proper amount of VASER in both the superficial and deep layers of fat. This allows for ease of removal of fat and most importantly limits the dreaded postoperative complication of contour irregularities.

For the lower back, it is imperative that we remove the deep compartment of fat. This fat is typically larger and more yellow in appearance than the superficial layer. By removing this, it can help achieve a more dynamic curve that individuals are seeking. In addition, it will help achieve a slight buttock lift.

Dr Duncan:

See the previous section. For patients with severe rectus diastasis, I pre-treat with Emsculpt (BTL Aesthetics, Boston, MA).

### Arms

Dr Pozner and Dr Cook:

Very easy to accomplish under local anesthesia with the BodyTite 40-watt handpiece, placing small incisions at the elbow and axillary fold. The awake patient is able to help you with positioning.

Dr DiBernardo and Dr Turer:

Although we have used all the tightening technologies, SmartLipo has done very well over the last 10 years with an excellent combination of fat reduction and tightening in patients even in their 60s.

Dr Kortesis and Dr Bharti:

Make sure to use a 3-mm cannula during liposuction and to go 360°. Avoid over resection and maintain standard liposuction techniques. This will help avoid the “shark bite” effect that results from over resection in certain areas and allow for a more uniform result.

Dr Duncan:

Be careful not to liposuction too aggressively or superficially, as this will leave cannula marks.

### Medial Thighs

Dr Pozner and Dr Cook:

We still use BodyTite so far, and believe this to be the best (until someone convinces me otherwise).

Dr DiBernardo and Dr Turer:

Very difficult area that always needs tightening or else a medial thigh lift. It is hard to get good transition planes. Always do anterior and posterior approaches.

Dr Kortesis and Dr Bharti:

Stay superficial in the medial thighs. Again, here we prefer a smaller cannula such as a 3 mm to avoid irregularities and deformities.

Dr Duncan:

This is a problem area in many cases. I tend to prefer a bipolar approach here (ie BodyTite). “Ironing” the skin using BodyTite can reduce crepiness.

### Knees and Lower Legs

Dr Pozner and Dr Cook:

Given the small surface areas, we prefer BodyTite in these areas.

Dr DiBernardo and Dr Turer:

Knees are one of the hardest areas to get good skin tightening, and it is important to explain to the patient that it will never be perfect. We approach the knees from four different incisions and have currently been doing the best with the FaceTite handpiece. Calves are another very difficult area. The area is too small and too tight to go in with larger cannulae. You need to first break up or melt the fat really well using a small diameter tool. We use SmartLipo because it is 1 mm, and we follow that up with a small diameter cannula for suction; no VASER (it is too big). Treat circumferentially and sculpt the calf muscles as needed. A word of caution about long-term swelling that may persist for up to 6 months. Good long-term compression is a must.

Dr Kortesis and Dr Bharti:

Be very careful to avoid over-resection in these areas. Use a smaller cannula (such as a 3 mm). We prefer a 360° approach for complete contouring and allowing the skin to re-drape evenly.

Dr Duncan:

For the knees, I use Renuvion or FaceTite, as the BodyTite handpiece is too powerful. For the lower leg, there must be enough fat to make a difference. In many cases, I will use Emsculpt for calves.

8. **What is your approach to secondary liposuction cases**? **To other special circumstances**?

Dr Pozner and Dr Cook:

We do a lot of secondary liposuction in our practice, and effective utilization of the basket cannula is key to success. We perform separation (following the SAFELipo technique^2^) during tumescent infiltration, using basket cannulas that are designed with a Luer lock for attaching our fluid infiltration tubing (Black & Black Surgical, Tucker, GA). We also find that using VASER really helps with fibrotic, scarred areas after previous liposuction. BodyTite is also very helpful to achieving skin tightening.

Dr DiBernardo and Dr Turer:

In secondary liposuction, there is a lot of scar tissue that, if not broken up, will leave worse dimpling. It needs to be divided or softened. VASER helps a bit with this, but the 1440 nm has a very high affinity for water. We use the side-firing Cellulaze (Cynosure) 1000u fiber to break this up. Surface scar tissue is a topic for another round table discussion.

Dr Kortesis and Dr Bharti:

Secondary liposuction cases can be very difficult. VASER is a must in the cases, and we really spend a lot of time to use the VASER to break down adhesions and scar tissue. We then also spend a fair amount of time performing separation and fat equalization. In these cases, the amount of time spent on the actual liposuction part should be minimal in comparison. In cases with significant scar tissue, our goal is to use these techniques to make it easy for the liposuction cannula to pass, before actually performing liposuction. This will decrease and potentially eliminate any issues of deformities.

Dr Duncan:

Secondary liposuction is a tough subject. I have done some Renuvion in regions with residual laxity as the device is good in the more fibrotic regions (flanks and bra roll). However, in a secondary neck, I would not use Renuvion, as the gas could possibly be driven into the mediastinum because of the resistance of previously created scar tissue. Clinically, it is important to take a good look at the previously treated areas beforehand. If the tissue is stiff, unyielding, and fibrotic, treatment with BodyTite or Renuvion is contraindicated. There would be no clinical benefit. Treatment of fatty laxity could be performed in areas, but I would avoid scar tissue.

9. **Do you combine liposuction with excisional procedures (such as abdominoplasty, brachioplasty, or thigh lift)**?

Dr Pozner and Dr Cook:

We do combine liposuction with abdominoplasty in appropriately selected patient, but it is important to remember that Florida has rules on how much liposuction can be performed in conjunction with another procedure. When combining liposuction with brachioplasty, it prolongs recovery. Similarly, liposuction can be combined with a thigh lift, but it is important to be careful not to overdo the resection.

Dr DiBernardo and Dr Turer:

We will combine liposuction with abdominoplasty but use smaller areas of dissection. We often combine liposuction with brachioplasty if the excision is very large; in these cases, we do six laser scar treatments afterward. With a thigh lift, we will perform liposuction if there is a large anterior component, but be careful: if there is loose skin, this could make it look worse.

Dr Kortesis and Dr Bharti:

We believe it is imperative when doing excisional procedures that liposuction be a part of the operation. The extent of the amount of adiposity in a given area will determine the amount of liposuction. We believe in full concurrent liposuction followed by the excisional operation. This allows for ultimately better results and outcomes.

Dr Duncan:

I do combine liposuction with excisional procedures, in moderation. The character of the residual tissue tends to be flabby, with a poor soft tissue scaffold or framework due to intrinsic collagen atrophy within the fatty layer. Age-related histology and scanning electron microscopy (SEM) show a proportional decline in the stromal/vascular component of tissue as people age. By rebuilding the collagen network with multilevel RF, that scaffold can be restored, giving a firm feel and defined shape to the soft tissue.

When I combine liposuction with abdominoplasty, I perform liposuction centrally, especially peri-umbilically where laxity and striae are prominent. During brachioplasty, I perform circumferential liposuction in unresected regions. And for thigh lift, I believe liposuction helps residual tissue achieve tone and tightness.

10. **What are your preferences for postoperative care**?

Dr Pozner and Dr Cook:

Our preference is for very limited use of postoperative compression garments—typically only for a few days postoperatively. We have seen a lot of problems with creasing and contour irregularities that seem to result from the garments “riding down.” We use Lipo Foam (Contour MD, Lenexa, KS) under the garments for large areas of liposuction (such as the abdomen or back), and we believe it can reduce these creases and possibly improve contour. We do not use drains after liposuction. We close incisions with simple interrupted Proline sutures.

Dr DiBernardo and Dr Turer:

We recommend 3-4 weeks of compression garments, to help mold and reduce swelling. We do not use drains, except perhaps above the buttocks. We close our liposuction incisions lightly. We instruct our patients to apply Alastin transform (Alastin Skincare, Carlsbad, CA) to areas of tightening twice a day.

Dr Kortesis and Dr Bharti:

We believe postoperative garments are imperative. We use Topifoam (Mentor Aesthetics, Santa Barbara, CA) with compression garments for 3 weeks, then followed by Spanx (Atlanta, GA) or other secondary garments for one additional month. We believe that drains are mandatory for the abdomen and back unless you leave incisions open to drain out. For large volume areas such as the abdomen and back, we prefer drains because of the excess amount of fluid that those areas produce. For extremities (arms and legs), some incisions are left open to allow for faster recovery and less postoperative swelling. Because we leave drains, incisions are closed for the abdomen and trunk and left open for extremities.

Proper nutrition and fluid management are essential in these cases. Patients are encouraged to drink 1 ounce of water per kilogram body weight per day. In addition, patients are encouraged to increase their protein intake to help with the recovery phase. We have lymphatic massage starting week 1 and then Endermologie (LPG, Valence, France) starting in week 3 to help with swelling and potential irregularities.

Dr Duncan:

I strongly encourage the wearing of a compression garment during healing. For previously pendulous areas, I will add Topifoam and KT tape. I always use drains for abdominoplasties but never for liposuction-based procedures (I use compression instead). I close my incisions; it looks nicer, but not so tight that fluid or gas cannot drain through. Finally, I limit activity for the first week to light activities of daily living.

11. **What are the future directions in liposuction**? **Upcoming treatment procedures/approaches, or future technologies**?

Dr Pozner and Dr Cook:

We are not aware of any new upcoming surgical technologies, but we are working on several nonsurgical technologies for body contouring. We have been using LIPOcel (Jeisys, Seoul, South Korea) vs the eon Laser (Dominion Aesthetic Technologies, San Antonio, TX) for adipolysis. We are starting to use Sofwave (Sofwave Medical, Tustin, CA), a high-intensity-focused ultrasound device, for skin tightening. We have also been using the Infini Genius RF microneedling device (Lutronic, Billerica, MA) for abdominal skin tightening and have been impressed with the results.

Dr DiBernardo and Dr Turer:

The future is the progress of noninvasive technologies and targeting all three layers of tissue: skin, fat, and muscle. Upcoming devices that I am excited about include: Evolve and Evoke (Inmode, Lake Forest, CA), Sofwave, LIPOcel, and the eon Laser (Dominion Aesthetic Technologies). The big breakthrough in the future will be that some of these technologies will be able to do both fat reduction and skin tightening.

Dr Kortesis and Dr Bharti:

We will continue to advance the level of liposuction. More 3D and 4D results will be desired and be able to be achievable. Technologies will allow for less need for excisional procedures.

Dr Duncan:

Two treatment adjuncts that I will mention are Collagenase (Endo Pharmaceutical, Malvern, PA) and Emtone (BTL Aesthetics, Boston, MA). Endo Pharmaceutical has developed a collagenase product (Qwo, Endo Aesthetics, Malvern, PA) that reduces cellulite, and an off-label use is for post-liposuction deformities. I find that it works well for fibrosis, tethered scarring, and contour depressions. Another significant problem that I see is in patients with skin laxity, striae, and pendulousness. If fat is removed, skin quality tends to worsen in patients with thin, inherently crepey skin. Areas of concern tend to be volar arms, periumbilical abdomen, and inner thighs. Emtone uses shock waves and HIFEM to noninvasively improve skin tone.

The above technologies are currently available, but the future might bring advancements that are biological. Exosomes, nanofat, and similar derivatives are already being investigated. In the future, it may be possible to bank and process your own fat and exosomes, so that only a single donor event is needed.
